# Differential effects of lifetime stressors on major depressive disorder severity: a longitudinal community-based cohort study

**DOI:** 10.1192/j.eurpsy.2024.1783

**Published:** 2024-10-04

**Authors:** Yingying Su, Muzi Li, Jean Caron, Daqi Li, Xiangfei Meng

**Affiliations:** 1 School of Public Health and Emergency Management, Southern University of Science and Technology, Shenzhen, China; 2 Department of Psychiatry, Faculty of Medicine and Health Sciences, McGill University, Montreal, QC, Canada; 3 Division of Mental Health & Society, Douglas Research Centre, Montreal, QC, Canada; 4 Interdisciplinary School of Health Sciences, Faculty of Health Sciences, University of Ottawa, ON, Canada; 5 Mental Health Center, University-Town Hospital of Chongqing Medical University, Chongqing, China

**Keywords:** general psychopathology, lifetime stressors, longitudinal data, major depressive disorder, subtypes

## Abstract

**Background:**

Stressors across the lifespan are associated with the onset of major depressive disorder (MDD) and increased severity of depressive symptoms. However, it is unclear how lifetime stressors are related to specific MDD subtypes. The present study aims to examine the relationships between MDD subtypes and stressors experienced across the lifespan while considering potential confounders.

**Methods:**

Data analyzed were from the Zone d’Épidémiologie Psychiatrique du Sud-Ouest de Montréal (*N* = 1351). Lifetime stressors included childhood maltreatment, child–parent bonding, and stressful life events. Person-centered analyses were used to identify the clusters/profiles of the studied variables and multinomial logistic regression analyses were performed to examine the relationships between stressors and identified MDD subtypes. Intersectional analysis was applied to further examine how distal stressors interact with proximal stressors to impact the development of MDD subtypes.

**Results:**

There was a significant association between proximal stressors and melancholic depression, whereas severe atypical depression and moderate depression were only associated with some domains of stressful life events. Additionally, those with severe atypical depression and melancholic depression were more likely to be exposed to distal stressors such as childhood maltreatment. The combinations of distal and proximal stressors predicted a greater risk of all MDD subtypes except for moderate atypical depression.

**Conclusions:**

MDD was characterized into four subtypes based on depressive symptoms and severity. Different stressor profiles were linked with various MDD subtypes. More specific interventions and clinical management are called to provide precision treatment for MDD patients with unique stressor profiles and MDD subtypes.

## Introduction

### Major depressive disorder and its subtypes

Major depressive disorder (MDD) is a common but heterogeneous mental disorder. According to the World Health Organization (WHO) report, 322 million people suffered from MDD which accounted for 4.4% of the global population resulting in a surge in suicide rates as well as a huge social and economic burden [1]. A diagnosis of MDD in the Diagnostic and Statistical Manual of Mental Disorders (DSM) criteria 5^th^ edition is made if 5 out of 9 symptoms are met, and several of these symptoms are opposites (e.g., weight gain or loss), two people with the same MDD diagnosis may have few symptoms in common [2]. Although DSM-5 contains several specifiers such as depression with atypical or melancholic features, they have not proved sufficient to predict prognosis and treatment responses [3, 4]. Because the diagnosis of MDD does not reflect the possible combinations of depressive symptoms, the heterogeneity of MDD significantly hinders the advancement in the etiology and treatment options [5, 6]. This heterogeneity underscores the necessity of an in-depth investigation of clinical symptoms and relevant risk factors [7].

A different approach to examining the heterogeneity of MDD is through person-based methods, such as latent class analysis (LCA) to identify symptoms that tend to co-occur in patient subgroups [8]. These techniques cluster patients based on the congregation of different depressive symptoms without a pre-conceived hypothesis. Studies have identified an “atypical” subtype characterized by increased sleep and appetite, a “typical” or “melancholic” subtype characterized by decreased sleep and appetite, and the presence of psychomotor symptoms [9–11]. These subtypes and their most distinguishing symptoms such as appetite and weight gain or loss were also linked with distinct biological and genetic correlates and different neural activities [12, 13]. Particular pharmacological interventions may be indicated in some subtypes (e.g., monoamine oxidase inhibitors in atypical depression) highlighting their potential clinical utility [14]. However, some promising exceptions exist. Previous studies have yielded inconclusive findings between the increase and decrease in sleep, appetite, weight, and psychomotor symptoms in various population groups, while these distinctions may be crucial in identifying MDD subtypes [9, 10, 15]. Likewise, studies pointed out that the DSM melancholic or atypical specifiers cannot help to create more homogeneous MDD subgroups [16, 17]. To further examine the validity, potential etiological attributes, and clinical usefulness of these subtypes, it is essential to analyze how specific depressive symptoms inform the research of etiology, diagnosis, and the development of targeted treatment [18].

### Stressors are known for their increased risk of developing MDD

Exposure to lifetime stressors and their related stress responses have been the focus of substantial MDD research [19–21]. One main reason to study stress in MDD is that adverse childhood experiences and chronic adulthood stressors are common experiences that portend the development of a variety of costly and burdensome mental and physical health outcomes [22–24]. According to the diathesis-stress theory, exposure to stressors may activate a diathesis or vulnerability, transforming the potential or predisposition into the actuality of psychopathology [25]. Exposure to early life adversities and adverse stressful life events serve as general vulnerability factors that make individuals more sensitive to stress challenges and therefore can feed forward into exacerbation of ongoing, or greater susceptibility toward future stress-related disease states, especially as they pertain to negative affect and mental health in general [26–28].

Although evidence has suggested that the differential effects of stressors on MDD vary across different stages of life [29–31], little is known about which stressor has the most influential impact on MDD subtypes by contrasting stressors at different life stages, various stressors profiles across the lifespan, and proximal vs. distal stressors. Even less is learned about how these different measures of stressors intersect to increase overall vulnerability to MDD subtypes. Identification of the distinct roles of stressors may be essential in determining the biological bases of depression [32, 33]. Treatment guidelines and procedures could then be more personalized when individuals with stressors and those without such stressors but with the same diagnostic labels are differentiated.

To the best of our knowledge, no studies have been conducted or published to comprehensively articulate the complex relationships between stressors across the lifespan and MDD subtypes. In the present study, we aim to untangle the relationships between MDD subtypes and various stressor measures across the lifespan and to explore how stressors intersect in MDD subtypes.

## Methods

### Study sample

**Figure 1.**
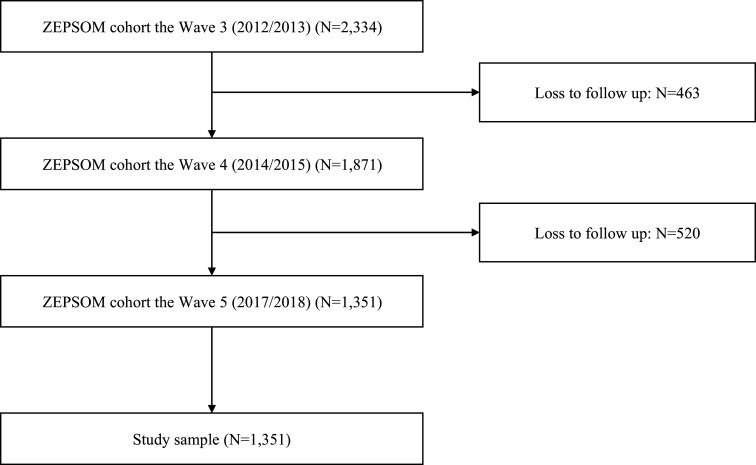
Process of the study sample selection.

Data were from the ongoing longitudinal cohort Zone d’Épidémiologie Psychiatrique du Sud-Ouest de Montréal (ZEPSOM). It is a community-based population cohort randomly selected from Montreal Southwest, Canada. In 2007, a sample of 2433 participants aged 15 to 65 years was randomly recruited at baseline and the following data were collected at a two-year interval. More details about the study cohort could be found in the previous research [34]. Because this study focused on lifetime stressors and MDD, only those respondents (*N* = 1351) who had complete information on depressive symptoms at Wave 4, stressful life events at Wave 3, and early life adverse experiences at Wave 5, were selected for the present study. Figure 1 illustrates the process of the study sample selection.

### Measures

#### Distal and proximal stressors

The distal stressors were measured by the two following instruments:

##### Childhood maltreatment

Childhood maltreatment was assessed with the Childhood Trauma Questionnaire-Short Form (CTQ-SF) [35]. It is a 28-item self-report inventory developed to measure five types of abuse or neglect including emotional abuse, physical abuse, sexual abuse, emotional neglect, and physical neglect in childhood or adolescence. The Cronbach’s alpha value was 0.98.

##### *Parental-child* bonding

Parental-child bonding was measured with the Parental Bonding Instrument (PBI) which is a 50-item self-administered questionnaire retrospectively measures children’s perceptions of parent-child relationships in terms of parental behaviors and attitudes before the age of 16 years [36]. The questionnaire has two subscales with each consisting of 12 items for assessing care dimension and 13 items for assessing overprotection and control dimension on both maternal and paternal sides. The Cronbach’s alpha value was 0.89 for the maternal care scale and the 0.93 for paternal care scale, and 0.84 for both the maternal and the paternal overprotection subscales.

##### Stressful life events

Stressful life events that occurred in the past 12 months prior to the data collection (as a proxy of proximal stressors) were measured with the Life’s Events Questionnaire [37]. It includes 22 items assessing five themes including work and financial issues, romantic relationships, links with family and friend relationships, housing, and experiences of aggression. Its Cronbach’s alpha value in the present study was 0.58.

#### Depressive symptoms

MDD was assessed using the World Mental Health Survey Composite International Diagnostic Interview (WMH-CIDI), a fully structured diagnostic interview based on the criteria of the DSM- 4th edition and the International Statistical Classification of Diseases and Related Health Problems, 10^th^ revision (ICD-10) [38, 39]. Nine DSM-4 depressive symptoms (including depressed mood, weight loss/gain, increased/decreased appetite, insomnia/hypersomnia, and psychomotor agitation/retardation) listed in the WMH-CIDI were used in the study. We separated these symptoms into a total of 17 symptoms, including sadness, nothing rejoicing, discouragement, pessimism about future, loss of interest, decreased appetite, increased appetite, weight loss, weight gain, insomnia, hypersomnia, psychomotor retardation, psychomotor agitation, fatigue, worthlessness/guilt, poor concentration and suicidal thoughts.

#### Covariates

Factors, including age, sex, ethnicity, marital status, education attainment, income, immigration status, family history of mental disorders, and comorbidities in mental illnesses were also included. Age was split into four age subgroups: 18 to 29 years, 30 to 44 years, 45 to 59 years, and 60 years and above. Marital status was categorized into single, married/common-law, and separated/divorced/widowed. Education attainment was grouped as less than junior education, high school graduation, and post-high school. Four categories of personal income were generated: less than $10,000, $10,000 to $29,999, $30,000 to $59,999, and $60,000 or more. Respondents were also asked about their family history of mental disorders whether a biological parent, or both, had been diagnosed by a psychiatrist, or hospitalized or experienced any of the following mental health problems including depression, anxiety disorders, delirium or hallucination, substance abuse, and suicide. Comorbidities in mental illnesses were dichotomized into with and without subgroups based on the answers on whether the respondent had any common mental disorder including generalized anxiety disorder, panic disorder, social phobia, agoraphobia, post-traumatic stress disorder, alcohol abuse and dependence, or drug abuse and dependence.

#### Statistical analysis

Person-centered approaches were used to identify MDD subtypes and stressors profiles across the lifespan. LCA was first used to discover MDD subtypes based on their responses to the WMH-CIDI depressive symptoms. The optimal number of latent classes was determined by the model fit among the four consecutive models with 2-5 classes each, based upon fit indices under the following conditions: lower Akaike Information Criterion (AIC), lower Bayesian Information Criterion (BIC), lower sample size adjusted BIC, higher entropy values, and a significant bootstrap likelihood ratio test (BLRT) [40]. To quantify the impact of stressors across the lifespan, two approaches were used including a composite stress index and cumulative stressors profiles using the latent profile analysis (LPA) [29]. Similarly, the optimal number of latent stressor profiles was determined by the above-mentioned procedure. The relationships between various stressors measures across the lifespan (the composite stress index, cumulative stressors profiles, childhood maltreatment, parent-child bonding, and stressful life events) and MDD subtypes were then assessed by multinomial logistic regressions considering those covariates (age, sex, ethnicity, marital status, education attainment, income level, immigration status, family history of mental disorders and comorbidities in mental illnesses).

To have an in-depth understanding of how distal and proximal stressors intersected and interacted in different MDD subtypes, we also compared the independent and combined effects of distal or/and proximal stressors across each MDD subtype. The predicted probabilities of each MDD subtype were calculated to estimate the MDD probability an individual exposed to a certain pattern of stressors would have.

Descriptive analysis and regression models were performed using Stata, version 15, and the LPA and LCA were conducted using Mplus, version 8. Significance was set at a 2-sided *P* < 0.05.

## Results

The sociodemographic characteristics of the study samples are presented in Supplementary Table S1. Overall, 63.1% were females and aged 45 or more. Less than half of them were single (44.2%). Most study subjects were white (87.3%) and non-immigrants (80.9%). Approximately two-thirds of them reported receiving a post-high school education (65.4%) and earned more than $30,000 annually (67.8%). A total of 9.7% (*N* = 131) of the study sample were diagnosed with past 12-month MDD at Wave 4 in the current study.

### Latent classes of depression subtypes

**Figure 2.**
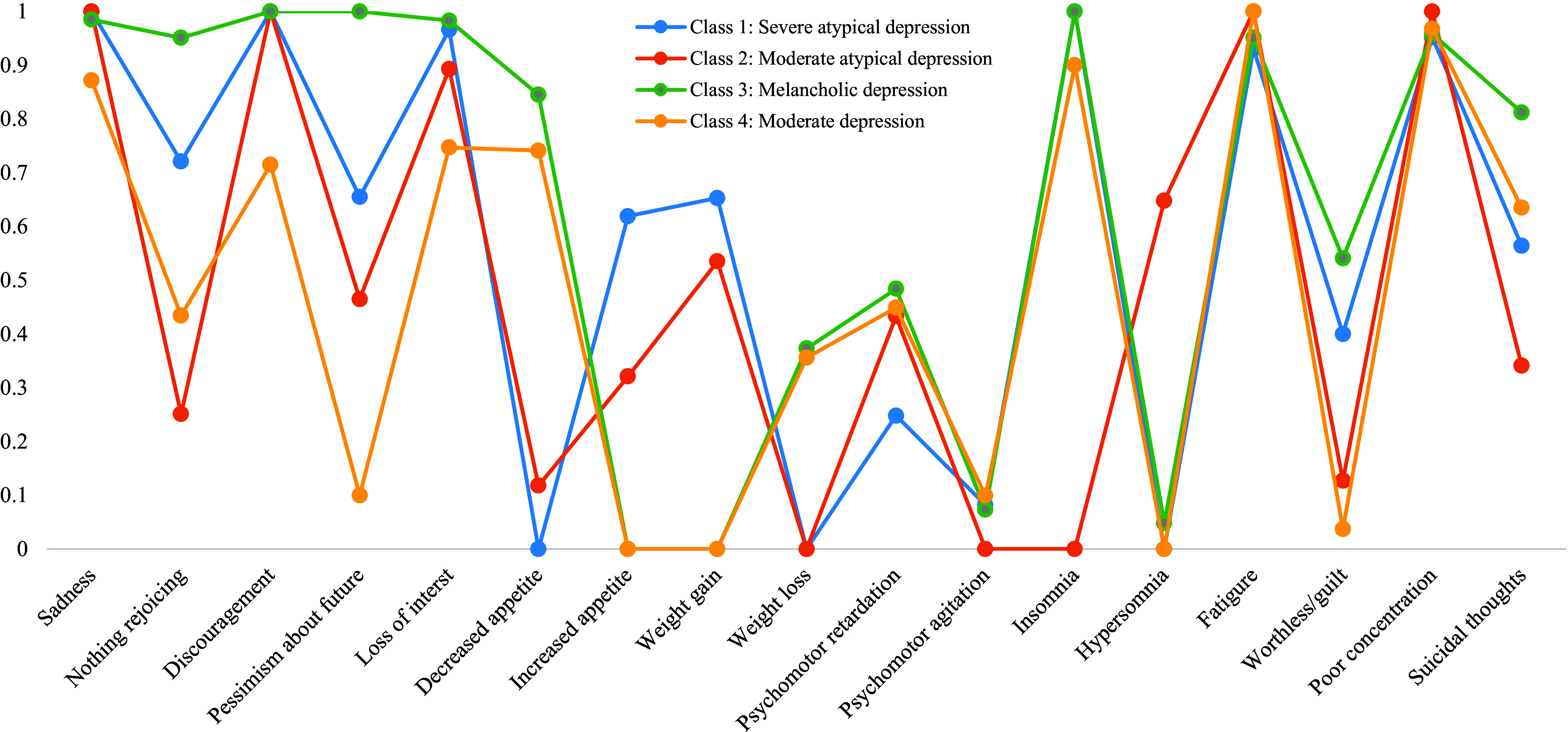
Latent profiles of major depressive disorder subtypes based on depressive symptoms.

The 4-class model of MDD subtypes with the best model fit was chosen for subsequent analyses. BLRT showed no statistical significance between the 4-class model and the 5-class model, indicating that there was no improvement in model specification from the 4-class model to the 5-class model. Details of the model fit indices of different latent class models are presented in Table 1. Figure 2 illustrates various combinations of the studied depressive symptoms based on the four-class LCA model. Class 3 was labeled as “Melancholic depression” for having high intra-class probabilities of depressed mood, decreased appetite, weight loss, insomnia, psychomotor retardation, poor concentration, feeling worthless, and thoughts of death. It was the most prevalent MDD subtype and had a prevalence of 46.5%. Class 1 was named “Severe atypical depression,” which was marked by increased appetite and weight gain. The prevalence of Class 1 was 22.2%. Class 2 (prevalence: 7.1%) was labeled as “Moderate atypical depression” since it had lower probabilities of atypical depressive symptoms than the “Severe atypical depression” group. Class 4 (prevalence: 24.1%) was labeled as “Moderate depression” owing to its less pronounced and moderately severe depressive symptoms of a typical symptom pattern characterized by decreased appetite, weight loss, and insomnia.

**Table 1.** Model fit indices for a two-class, three-class, four-class, and five-class solutions of major depressive disorder subtypes

Number of classes	2	3	4	5
AIC	1844	1779	1773	1775
BIC	1945	1931	1977	2032
ssaBIC	1834	1763	1753	1750
Entropy	0.87	0.87	0.94	0.95
Log–likelihood	–960	–887	–837	–815
Difference in log–likelihood	<0.01	<0.01	<0.01	0.09

Abbreviations: AIC, Akaike’s information criteria; BIC, Bayesian information criteria; ssaBIC, the sample size adjusted Bayesian information criteria.

### Latent profiles of lifetime stressors

Similarly, a 3-profile model was selected for the lifetime stressors based on the model fit indices (see Table 2). A total of 75.1% participants reported a low level of lifetime stressors, thus was labelled as the “Low stressor” group. There were 6.8% and 18.1% of participants who had scores of high and moderate levels of stressors and were labelled as “High stressor” and “Moderate stressor” groups, respectively.

**Table 2.** Model fit indices for a one-class, two-class, three-class, and four-class solution for lifetime stressors

Number of profiles	1	2	3	4
AIC	78,442	75,529	74,353	73,447
BIC	78,580	75,743	74,641	73,810
ssaBIC	78,492	75,606	74,457	73,578
Entropy	—	0.95	0.97	0.91
Log– likelihood	—	–39,193	–37,721	–37,118
Difference in log–likelihood	—	<0.01	<0.01	<0.01

Abbreviations: AIC, Akaike’s Information Criteria; BIC, Bayesian Information Criteria; ssaBIC, the sample size adjusted Bayesian Information Criteria.

*Note*: Lifetime stressors considered childhood maltreatment, child–parent bonding, and stressful life events.

### Associations between different stressor measures and MDD subtypes

For early life stressors, the multinomial regression model showed that emotional abuse (OR, 1.11, 95 % CI, 1.02–1.20), sexual abuse (OR, 1.09, 95 % CI, 1.01–1.18), physical neglect (OR, 1.20, 95 % CI, 1.08–1.33) and emotional neglect (OR, 1.09, 95 % CI, 1.00–1.19) were significantly independent risk factors for Severe atypical depression, but not for Moderate atypical depression (*p* ≥ 0.05) after controlling for age, sex, ethnicity, marital status, education attainment, income level, immigration status, family history of mental disorders and comorbidities in mental illnesses. Likewise, Melancholic depression was associated with a history of childhood maltreatment including physical abuse (OR, 1.10, 95 % CI, 1.02–1.18), emotional abuse (OR, 1.12, 95 % CI, 1.05–1.19), physical neglect (OR, 1.13, 95 % CI, 1.04–1.24) and emotional neglect (OR, 1.10, 95 % CI, 1.03–1.17). However, only emotional abuse was found to be significantly associated with Moderate depression (OR, 1.10, 95 % CI, 1.01–1.20). There was no significant relationship between different dimensions of parent-child bonding and depression subtypes, except that a weak association was found between less maternal care and Melancholic depression (OR, 1.01, 95 % CI, 1.00–1.03).

With regards to the later-on stressful life events, all the five domains of life events were significant risk factors for “Melancholic depression.” Individuals who had work and financial issues, romantic relationship-related stressors, family/friend relationship-related stressors, housing issues, experience of aggression had 1.13 (95 % CI, 1.04–1.22), 1.38 (95 % CI, 1.20–1.60), 1.21 (95 % CI, 1.12–1.31), 1.29 (95 % CI, 1.10–1.51), and 1.22 (95 % CI, 1.13–1.58) times the risk of developing “Melancholic depression,” respectively. Work and financial issues (OR, 1.15, 95 % CI, 1.03–1.27), family/friend relationship-related stressors (OR, 1.17, 95 % CI, 1.04–1.31) and experience of aggression (OR, 1.49, 95 % CI, 1.11–1.98) increased the risk of “Moderate depression,” while the remaining two domains, romantic relationship related stressors (OR, 1.31, 95 % CI, 1.07–1.60) and housing issues (OR, 1.31, 95 % CI, 1.07–1.60) increased the risk of “Severe atypical depression.”

Cumulative stressors increased the risk for “Severe atypical depression” (OR, 1.12, 95 % CI, 1.05–1.19), “Melancholic depression” (OR, 1.12, 95 % CI, 1.07–1.18), and “Moderate depression” (OR, 1.11, 95 % CI, 1.03–1.18). For different stressor profiles, compared to individuals with a “Low stressor” level, individuals with the level of “Moderate stressor” and the level of “High stressor” were 2.83 times (95% CI, 1.04–7.40) and 3.17 times (95% CI, 1.61–6.24) more likely to develop “Melancholic depression,” respectively. Moreover, those in the “High stressor” group were associated with an increased risk of “Severe atypical depression” (OR, 2.82, 95% CI, 1.08–7.36) and “Moderate depression” (OR, 3.39, 95% CI, 1.10–8.20), respectively. In contrast, no significant associations between different stressor measurements and “Moderate atypical depression” were found (see Table 3). All the above-mentioned results are based on the analyses adjusted for covariates.

**Table 3.** Associations between different psychosocial stress measures and major depressive disorder subtypes*

Stressors	Severe atypical (*N* = 300)	Moderate atypical (*N* = 96)	Melancholic (*N* = 629)	Moderate (*N* = 326)
	OR (95% CI)	OR (95% CI)	OR (95% CI)	OR (95% CI)
*Individual psychosocial stress*				
Physical abuse	1.09(0.98–1.21)	0.89(0.65–1.22)	**1.10(1.02–1.18)**	1.06(0.94–1.18)
Emotional abuse	**1.11(1.02–1.20)**	0.77(0.51–1.15)	**1.12(1.05–1.19)**	1.08(0.98–1.18)
Sexual abuse	**1.09(1.01–1.18)**	0.62(0.36–1.07)	1.04(0.96–1.12)	1.02(0.92–1.14)
Physical neglect	**1.20(1.08–1.33)**	0.83(0.54–1.29)	**1.13(1.04–1.24)**	1.08(0.94–1.24)
Emotional neglect	**1.09(1.00–1.19)**	0.93(0.77–1.13)	**1.10(1.03–1.17)**	**1.10(1.01–1.20)**
Maternal care	1.01(0.99–1.03)	0.93(0.83–1.05)	**1.01(1.00–1.03)**	1.02(0.99–1.04)
Maternal control	0.97(0.94–1.00)	1.05(0.93–1.18)	0.97(0.94–1.00)	0.96(0.93–1.00)
Paternal care	1.00(0.98–1.02)	1.00(0.94–1.07)	1.01(0.99–1.02)	1.01(0.99–1.03)
Paternal control	0.99(0.95–1.02)	1.10(0.96–1.25)	0.97(0.95–1.00)	0.98(0.94–1.03)
Work and financial issue	1.05(0.92–1.20)	1.06(0.84–1.28)	**1.13(1.04–1.22)**	**1.15(1.03–1.27)**
Romantic relationship	**1.31(1.07–1.60)**	1.12(0.73–1.71)	**1.38(1.20–1.60)**	1.23(0.95–1.59)
Family/friend relationship	1.10(0.98–1.23)	0.88(0.68–1.13)	**1.21(1.12–1.31)**	**1.17(1.04–1.31)**
Housing	**1.31(1.07–1.60)**	0.48(0.19–1.23)	**1.29(1.10–1.51)**	0.87(0.62–1.23)
Experience of aggression	1.26(0.92–1.73)	1.09(0.56–2.10)	**1.22(1.13–1.58)**	**1.49(1.11–1.98)**
*Cumulative psychosocial stress*				
Cumulative stress	**1.12(1.05–1.19)**	0.92(0.77–1.09)	**1.12(1.07–1.18)**	**1.11(1.03–1.18)**
*LPA–derived psychosocial stress clusters*				
Low stress level	–	–	–	–
Moderate stress level	2.16(0.47–9.00)	1.49(0.80–12.01)	**2.83(1.04–7.40)**	0.95(0.27–3.33)
High stress level	**2.82(1.08–7.36)**	NA	**3.17(1.61–6.24)**	**3.39(1.10–8.20)**

Abbreviations: CI, confidence interval; LPA, latent profile analysis; OR, odds ratio.

p < 0.05.

* Adjusted for age, sex, marital status, ethnicity, education attainment, income level, immigration status, family history of mental disorders, and comorbidity in mental illnesses including generalized anxiety disorder, panic disorder, social phobia, agoraphobia, post-traumatic stress disorder, alcohol abuse, and dependence, as well as drug abuse and dependence.

*Note*: OR = Odds ratio; CI = Confidence interval; LPA = Latent profile analysis.

### Differences in associations between combinations of distal stressors and proximal stressors and major depressive disorder subtypes

**Figure 3.**
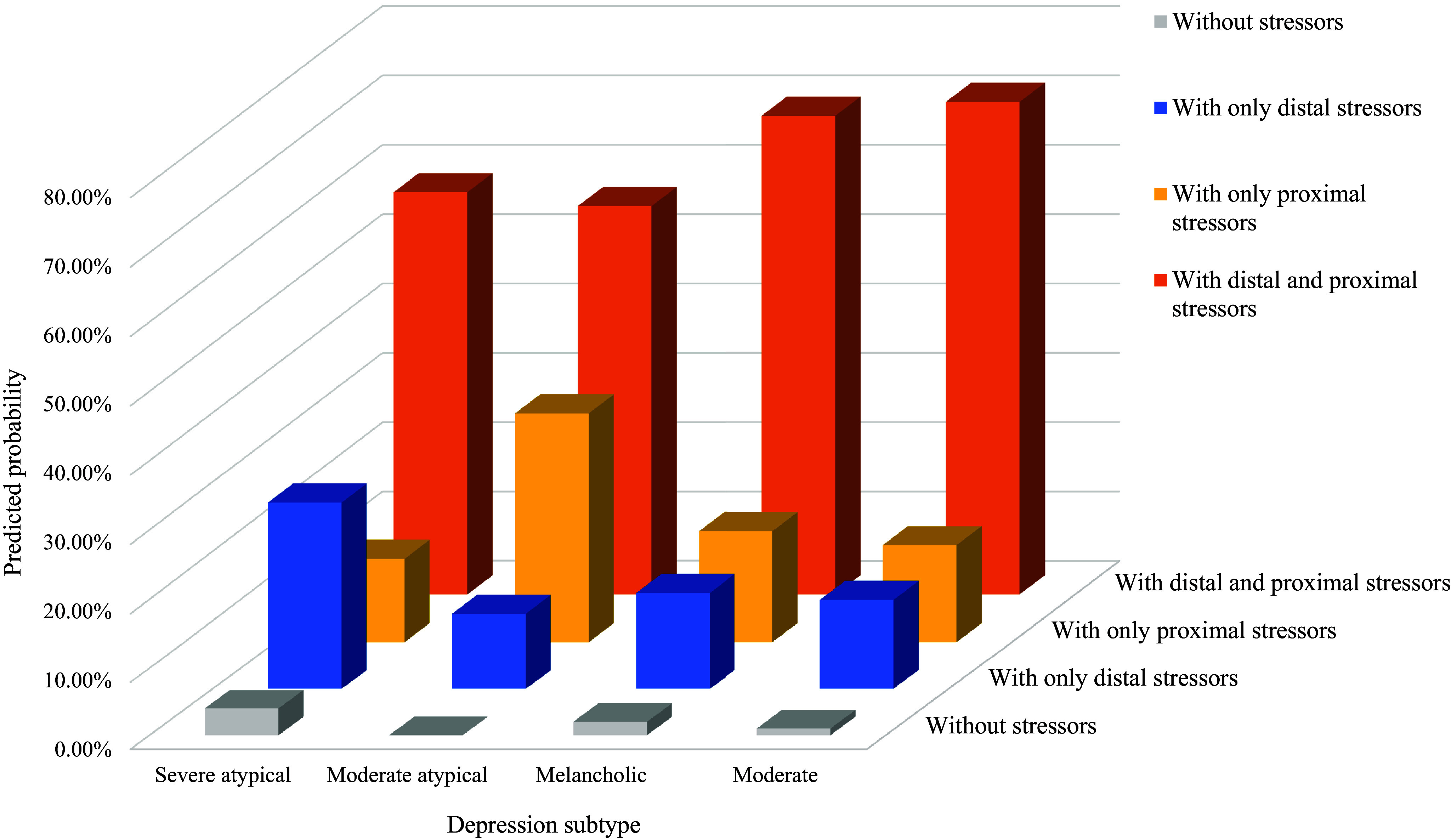
Predicted probability of major depressive disorder subtypes across different stressor profiles.

We further compared the intersectionality of proximal and distal stressors in MDD subtypes. Table 4 illustrates differences in associations between various combinations of distal stressors and proximal stressors in four MDD subtypes. Exposure to both distal and proximal stressors increased the risk of all MDD subtypes (p ≤ 0.05) except for “Moderate atypical depression” (see Figure 3 as well). Specifically, for “Severe atypical depression,” it was more likely to occur in the presence of distal stressors (p = 0.03), regardless of whether proximal stressors were present. The proximal and distal stressors were significantly associated with “Melancholic depression” (p = 0.01), separately. Additionally, the co-occurrence of both distal and proximal stressors may have a cumulative effect, resulting in an increased risk of “Melancholic depression.” However, there was no significant difference in “Moderate depression” for those only exposed to distal or proximal stressors.

**Table 4.** Differences in major depressive disorder subtypes risk for different stressor profiles

Depression subtypes	Stressor exposures	Without stressors	With only distal stressors	With only proximal stressors	With distal and proximal stressors
Severe atypical depression	Without stressors		+	–	+
	With only distal stressors	+		+	–
	With only proximal stressors	–	+		–
	With distal and proximal stressors	+	–	–	
Moderate atypical depression	Without stressors		–	–	–
	With only distal stressors	–		–	–
	With only proximal stressors	–	–		–
	With distal and proximal stressors	–	–	–	
Melancholic depression	Without stressors		+	+	+
	With only distal stressors	+		–	–
	With only proximal stressors	+	–		+
	With distal and proximal stressors	+	–	+	
Moderate depression	Without stressors		–	–	+
	With only distal stressors	–		–	–
	With only proximal stressors	–	–		+
	With distal and proximal stressors	+	–	+	

*Note*: + indicates statistically significant difference; – indicates no difference.

## Discussion

The present study provides one of the first pieces of evidence on the differential associations between MDD subtypes and various combinations of stressors across the lifespan in a prospective Canadian community-based cohort. The four MDD subtypes identified in the present study indicate that both symptom patterns and severity of depressive symptoms were sources of MDD heterogeneity. Overall, we found that participants with a level of “high stressor” were more likely to develop different MDD subtypes. Not all the studied stressors were consistently associated with MDD subtypes. Specifically, there was a significant association between proximal stressors (all five subdomains of stressful life events) and “Melancholic depression,” whereas “Severe atypical depression” and “Moderate depression” were only associated with some domains of stressful life events. When compared to depression-free participants, those with “Severe atypical and Melancholic depression” were more likely to have had exposure to distal stressors such as child maltreatment. The combinations of distal and proximal stressors predicted a greater risk of all MDD subtypes except for “Moderate atypical depression.” No specific stressor was associated with “Moderate atypical depression.”

Consistent with the recent review, MDD subtypes were generally differentiated by depression severity and unique combinations of probabilities of depressive symptoms [41]. “Melancholic depression” and “Atypical depression” were the common MDD subtypes identified in the review. We discovered four MDD subtypes with various combinations of depressive symptoms and symptoms severity, including “Melancholic,” “Severe atypical,” “Moderate atypical,” and “Moderate depression.” Although no direct comparison can be made for the discovered MDD subtypes across studies as different depressive symptoms considering in each study, the four MDD subtypes identified in the current study demonstrated heterogeneous clinical manifestations.

Stress is found to be a universal process shared by both physiological and psychological systems when people cannot manage the demands or are out of balance [42, 43]. Ample research has consistently shown that early life stressors, major stressful events, as well as their associated stress responses, can activate a cascade of neurochemical and/or inflammatory factors to affect limbic corticotropin-releasing hormone (CRH) functioning [44]. These changes are linked to alterations of growth/anti-apoptotic factors which in turn, affect neuroplasticity, ultimately leading to psychopathology, i.e., MDD [45]. Adversities in early life can also interfere with children’s normal development and initiate a cascade effect negatively influencing children’s health and functioning [46]. Individual variations in the stress responses can be explained by the stressors as well as the differential vulnerability theory, which argues that certain stressors increase a higher risk of mental disorders [47, 48]. It is important to specify how different stressors are linked to MDD. Some neurobiological studies have indicated that differences in MDD subtypes might exist in the presence of certain neurochemical disturbances [45]. Accumulating evidence has shown that MDD subtypes can vary with regard to the hypothalamic-pituitary-adrenal (HPA) axis activity, immune function, and treatment response [12]. For instance, “Melancholic depression” or “Typical depression” was related to CRH neuronal hypoactive HPA axis, whereas “Atypical depression” was aligned with down-regulated HPA activity [49]. Given the complexity of the neurobiological mechanisms involved in MDD subtypes, it is critical to pinpoint the specific associations between each MDD subtype and diversified stressor exposures.

We found significant associations between childhood maltreatment and severe atypical depression as well as “Melancholic depression.” This finding is in line with a review on depression and stress, which also concluded that individuals with a history of childhood abuse or neglect had a higher risk of having “Severe atypical depression” or “Melancholic depression” [50]. Heim found that changes in the HPA axis could mediate clinical manifestations of depressive symptoms as a response to childhood maltreatment [51]. A history of childhood maltreatment was associated with significantly higher cortisol reactivity and total cortisol exposure. It also showed an opposing pattern of response based on the severity of depression, that is, cortisol levels decreased substantively among people exposed to childhood maltreatment with severe depression [52]. We also found that emotional neglect predicted “Severe atypical depression,” “Melancholic depression,” and “Moderate depression” but not “Moderate atypical depression.” The higher level of unpleasantness in these MDD subtypes could account for this phenomenon since emotional neglect contributes most to the dimension of anhedonic depression, which can be reflected by the inability to feel pleasure or rejoice [53]. However, the independent effects of physical abuse and sexual abuse on MDD subtypes have been inconclusive [54, 55].

We also discovered that recent major stressful life events increase the risk of melancholic and severe atypical depression. Stressors related to romantic relationships independently increased the risk of “Severe atypical depression,” which manifests a high level of guilt. This finding is consistent with a large-population-based twin study, which found that when depressive symptoms occurred in relation to stressful events, guilt was most common after romantic breakups [56]. Further, the independent effect of housing stressors on severe atypical depression was also identified in our study. Since “Severe atypical depression” was associated with a disturbing sense of numbness and emptiness [57], it is intuitive that housing movement or eviction can be directly related to it. In the same vein, stressors related to work, finance, or family/friend issues predicted “Moderate depression,” which had a moderately depressed mood and higher rates of fatigue and insomnia. These symptom profiles were often found to be associated with stressful events regarding work stressors and family/friend strains, ultimately resulting in the occurrence of “Moderate depression” [58–60]. Harkness and Monroe suggested that those with severe melancholic depression were more likely to have non-severe stressful events prior to depression onset [61]. “Melancholic depression” appears to be an activation and persistence of the normal stress response and has increased post-challenge cortisol levels thus may be sensitized to specific stress [62]. Additionally, the endocannabinoid system may also play an important role in the etiology of “Melancholic depression.” Similarities between “Melancholic depression” and an endocannabinoid deficiency were found including their increased sensitization to stressors [63]. Although the association between experience of violent aggression and MDD has been well documented [64], this study provides further evidence to illustrate people with moderate depression and a higher probability of experiencing symptoms like suicidal thoughts, were more likely to report recent aggression experiences. Such experiences might explain the negative outcomes in terms of suicidal ideation [65].

As expected, we identified that exposures to both distal and proximal stressors substantively increased the risk of having severe atypical depression, melancholic depression as well as moderate depression. The diathesis-stress model suggests that the development of MDD is influenced by the interplay between a pre-existing diathesis, representing a susceptibility, and a later-on stressor can together activate or exacerbate the vulnerability to MDD [25]. Our research findings further expand the diathesis-stress model of MDD by indicating that distal and proximal stressors are associated with specific MDD subtypes. In part, this phenomenon could be explained by the fact the stress responses triggered by early life stressors increased stress sensitization and/or proliferation to proximal stressors and thus caused uniform consequences across different psychobiologic mechanisms leading to different MDD subtypes [66]. Different combinations of stressors may eventually end up with different MDD subtypes. The intersectional results of distal and proximal stressors support unique combinations of stressors associated with unique depression subtypes. Such findings bridge stress-related etiological findings with clinical management, especially pharmacological interventions had shown promising results in terms of some MDD subtypes being more sensitive to certain antidepressants (e.g., monoamine oxidase inhibitors in atypical depression) [67]. The potential clinical utility, as well as the practical importance of prevention and intervention, could benefit from more explicitly formulated and tailored strategies to address specific associations between stressors and MDD subtypes.

### Strengths and limitations

Built upon person-centered approaches and multiple measures of stressors, this present study discovers specific associations between various stressor exposures across the lifespan and MDD subtypes in a community-based population cohort. Although stressors predicted the increased risk of MDD, different stressors and their combinations were linked to specific MDD subtypes. By utilizing an intersectional approach and the life course perspective, we were able to pinpoint the differential vulnerability to MDD subtypes when facing various exposures to distal stressors and proximal stressors across the lifespan.

There are several limitations to be noted. First, the present study utilized self-report measures to assess various stressors that might be prone to response bias. However, all stressors were assessed by validated and widely used stressor measures, such as CTQ, PBI, and SLE. Second, our study sample had predominantly Caucasians drawn from a high-income country. The generalizability of our findings to other ethnic populations or other population settings needs to be tested. Finally, some MDD subtypes were less prevalent, such as “Moderate atypical depression.” The research findings on “Moderate atypical depression” and various stressors may be impacted by insufficient sample size. Future studies with a larger sample are warranted.

Overall, various stressor profiles across the lifespan demonstrated differential impacts on MDD subtypes, which were characterized by different symptom probabilities and severity. Combinations of distal and proximal stressors dramatically increased the most common MDD subtypes, therefore early identification, targeted prevention programs, and clinical management are urgently called to reduce the negative consequences of such exposures among the most vulnerable populations. Special attention should be paid to addressing differential vulnerability among unique associations between the stressor profiles and MDD subtypes. The findings of the study direct further research into the underlying stress-related neurobiological mechanisms involved in diversified clinical manifestations of MDD.

## Supporting information

Su et al. supplementary material Su et al. supplementary material

## Data Availability

The ZEPSOM data is not currently freely available to researchers in general due to ethical and data management requirements. Interested researchers can directly contact the Research Team at: xiangfei.meng@mcgill.ca.

## Supplementary material

The supplementary material for this article can be found at http://doi.org/10.1192/j.eurpsy.2024.1783.

## References

[1] Friedrich MJ. Depression is the leading cause of disability around the world. JAMA. 2017;317(15):1517. doi:10.1001/jama.2017.3826. 28418490

[2] American Psychiatric Association. Diagnostic and statistical manual of mental disorders. 5th ed. Washington, DC: American Psychiatric Association; 2013.

[3] Lojko D, Rybakowski J. Atypical depression: current perspectives. Neuropsychiatr Dis Treat. 2017;13:2447–56. 29033570 10.2147/NDT.S147317 PMC5614762

[4] Parker G, Fink M, Shorter E, Taylor MA, Akiskal H, Berrios G, et al. Issues for DSM-5: whither melancholia? The case for its classification as a distinct mood disorder. Am J Psychiatry. 2010;167(7):745–7. doi:10.1176/appi.ajp.2010.09101525. 20595426 PMC3733615

[5] Lux V, Kendler KS. Deconstructing major depression: a validation study of the DSM-IV symptomatic criteria. Psychol Med. 2010;40(10):1679–90. doi:10.1017/S0033291709992157. 20059797 PMC3010198

[6] Goldberg D. The heterogeneity of "major depression.” World Psychiatry. 2011;10(3):226–8. doi:10.1002/j.2051-5545.2011.tb00061.x. 21991283 PMC3188778

[7] Lynch CJ, Gunning FM, Liston C. Causes and consequences of diagnostic heterogeneity in depression: paths to discovering novel biological depression subtypes. Biol Psychiatry. 2020;88(1):83–94. doi:10.1016/j.biopsych.2020.01.012. 32171465

[8] Lubke GH, Muthen B. Investigating population heterogeneity with factor mixture models. Psychol Methods. 2005;10(1):21–39. doi:10.1037/1082-989X.10.1.21. 15810867

[9] Lamers F, de Jonge P, Nolen WA, Smit JH, Zitman FG, Beekman AT, et al. Identifying depressive subtypes in a large cohort study: results from the Netherlands study of depression and anxiety (NESDA). J Clin Psychiatry. 2010;71(12):1582–9. doi:10.4088/JCP.09m05398blu. 20673552

[10] Rodgers S, Grosse Holtforth M, Muller M, Hengartner MP, Rossler W, Ajdacic-Gross V. Symptom-based subtypes of depression and their psychosocial correlates: a person-centered approach focusing on the influence of sex. J Affect Disord. 2014;156:92–103. doi:10.1016/j.jad.2013.11.021. 24373526

[11] Kendler KS, Eaves LJ, Walters EE, Neale MC, Heath AC, Kessler RC. The identification and validation of distinct depressive syndromes in a population-based sample of female twins. Arch Gen Psychiatry. 1996;53(5):391–9. doi:10.1001/archpsyc.1996.01830050025004. 8624182

[12] Lamers F, Vogelzangs N, Merikangas KR, de Jonge P, Beekman AT, Penninx BW. Evidence for a differential role of HPA-axis function, inflammation and metabolic syndrome in melancholic versus atypical depression. Mol Psychiatry. 2013;18(6):692–9. doi:10.1038/mp.2012.144. 23089630

[13] Simmons WK, Burrows K, Avery JA, Kerr KL, Taylor A, Bodurka J, et al. Appetite changes reveal depression subgroups with distinct endocrine, metabolic, and immune states. Mol Psychiatry. 2020;25(7):1457–68. doi:10.1038/s41380-018-0093-6. 29899546 PMC6292746

[14] Trivedi MH, Rush AJ, Crismon ML, Kashner TM, Toprac MG, Carmody TJ, et al. Clinical results for patients with major depressive disorder in the Texas medication algorithm project. Arch Gen Psychiatry. 2004;61(7):669–80. doi:10.1001/archpsyc.61.7.669. 15237079

[15] Alexandrino-Silva C, Wang YP, Carmen Viana M, Bulhoes RS, Martins SS, Andrade LH. Gender differences in symptomatic profiles of depression: results from the Sao Paulo megacity mental health survey. J Affect Disord. 2013;147(1-3):355–64. doi:10.1016/j.jad.2012.11.041. 23246363

[16] Lorenzo-Luaces L, Buss JF, Fried EI. Heterogeneity in major depression and its melancholic and atypical specifiers: a secondary analysis of STAR*D. BMC Psychiatry. 2021;21(1):454 doi:10.1186/s12888-021-03444-3. 34530785 PMC8447832

[17] Fried EI, Coomans F, Lorenzo-Luaces L. The 341 737 ways of qualifying for the melancholic specifier. Lancet Psychiatry. 2020;7(6):479–80. doi:10.1016/S2215-0366(20)30169-3. 32445681

[18] Fried EI, Nesse RM. Depression sum-scores don’t add up: why analyzing specific depression symptoms is essential. BMC Med. 2015;13:72. doi:10.1186/s12916-015-0325-4. 25879936 PMC4386095

[19] Hammen C. Stress and depression: old questions, new approaches. Curr Opin Psychol. 2015;4:80–5.

[20] Hammen C. Risk factors for depression: an autobiographical review. Annu Rev Clin Psychol. 2018;14:1–28. doi:10.1146/annurev-clinpsy-050817-084811. 29328780

[21] Pego JM, Sousa JC, Almeida OF, Sousa N. Stress and the neuroendocrinology of anxiety disorders. Curr Top Behav Neurosci. 2010;2:97–117. doi:10.1007/7854_2009_13. 21309108

[22] Richardson S, Shaffer JA, Falzon L, Krupka D, Davidson KW, Edmondson D. Meta-analysis of perceived stress and its association with incident coronary heart disease. Am J Cardiol. 2012;110(12):1711–6. doi:10.1016/j.amjcard.2012.08.004. 22975465 PMC3511594

[23] Kivimaki M, Nyberg ST, Batty GD, Fransson EI, Heikkila K, Alfredsson L, et al. Job strain as a risk factor for coronary heart disease: a collaborative meta-analysis of individual participant data. Lancet. 2012;380(9852):1491–7. doi:10.1016/S0140-6736(12)60994-5. 22981903 PMC3486012

[24] Steptoe A, Kivimaki M. Stress and cardiovascular disease: an update on current knowledge. Annu Rev Public Health. 2013;34:337–54. doi:10.1146/annurev-publhealth-031912-114452. 23297662

[25] Monroe SM, Simons AD. Diathesis-stress theories in the context of life stress research: implications for the depressive disorders. Psychol Bull. 1991;110(3):406–25. doi:10.1037/0033-2909.110.3.406. 1758917

[26] Tsur N, Abu-Raiya H. COVID-19-related fear and stress among individuals who experienced child abuse: the mediating effect of complex posttraumatic stress disorder. Child Abuse Negl. 2020;110(Pt 2):104694. doi:10.1016/j.chiabu.2020.104694. 32900515 PMC7430290

[27] Berg MT, Simons RL, Barr A, Beach SRH, Philibert RA. Childhood/adolescent stressors and allostatic load in adulthood: support for a calibration model. Soc Sci Med. 2017;193:130–9. doi:10.1016/j.socscimed.2017.09.028. 28982528

[28] Ludwig L, Pasman JA, Nicholson T, Aybek S, David AS, Tuck S, et al. Stressful life events and maltreatment in conversion (functional neurological) disorder: systematic review and meta-analysis of case-control studies. Lancet Psychiatry. 2018;5(4):307–20. doi:10.1016/S2215-0366(18)30051-8. 29526521

[29] Su YY, D’Arcy C, Li M, O’Donnell KJ, Caron J, Meaney MJ, et al. Specific and cumulative lifetime stressors in the aetiology of major depression: a longitudinal community-based population study. Epidemiol Psychiatr Sci. 2022;31:e3. doi:10.1017/S2045796021000779. 35078547 PMC8851045

[30] Lippard ETC, Nemeroff CB. The devastating clinical consequences of child abuse and neglect: increased disease vulnerability and poor treatment response in mood disorders. Am J Psychiatry. 2020;177(1):20–36. doi:10.1176/appi.ajp.2019.19010020. 31537091 PMC6939135

[31] Teicher MH, Samson JA. Childhood maltreatment and psychopathology: a case for ecophenotypic variants as clinically and neurobiologically distinct subtypes. Am J Psychiatry. 2013;170(10):1114–33. doi:10.1176/appi.ajp.2013.12070957. 23982148 PMC3928064

[32] Mezulis A, Salk RH, Hyde JS, Priess-Groben HA, Simonson JL. Affective, biological, and cognitive predictors of depressive symptom trajectories in adolescence. J Abnorm Child Psychol. 2014;42(4):539–50. doi:10.1007/s10802-013-9812-2. 24158642 PMC3976682

[33] Carlson MW, Oshri A. Depressive symptom trajectories among sexually abused youth: examining the effects of parental perpetration and age of abuse onset. Child Maltreat. 2018;23(4):387–98. 29888624 10.1177/1077559518779755

[34] Su Y, Li M, D’Arcy C, Caron J, O’Donnell K, Meng X. To what extent do social support and mastery mediate the association between childhood maltreatment and depression? A sequential causal mediation analysis. Epidemiol Psychiatr Sci. 2022;31:e77. 36263598 10.1017/S2045796022000609 PMC9677445

[35] Bernstein D, Fink L. Manual for the childhood trauma questionnaire. New York: The Psychological Corporation; 1998.

[36] Parker G, Tupling H, Brown LB. A parental bonding instrument. Br J Med Psychol. 1979;52(1):1–10.

[37] Laurin I. Facteurs de risque de la condition de sans domicile fixe: comparaison d’une cohorte de nouveaux sans domicile fixe et d’une cohorte de domiciliés pauvres. Canada, Ottawa: National Library of Canada= Bibliothèque nationale du Canada; 2000.

[38] World Helath Organization WH. The ICD-10 classification of mental and Behavioural disorders: Clinical descriptions and diagnostic guidelines. Geneva: World Health Organization, https://apps.who.int/iris/handle/10665/37958; 1992.

[39] American Psychiatric Association. Diagnostic and statistical manual of mental disorders. 4th ed., Text Revision); 2000.

[40] Arminger G, Stein P, Wittenberg J. Mixtures of conditional mean-and covariance-structure models. Psychometrika. 1999;64(4):475–94.

[41] Ulbricht CM, Chrysanthopoulou SA, Levin L, Lapane KL. The use of latent class analysis for identifying subtypes of depression: a systematic review. Psychiatry Res. 2018;266:228–46. 29605104 10.1016/j.psychres.2018.03.003 PMC6345275

[42] Lupien SJ, McEwen BS, Gunnar MR, Heim C. Effects of stress throughout the lifespan on the brain, behaviour and cognition. Nat Rev Neurosci. 2009;10(6):434–45. 19401723 10.1038/nrn2639

[43] Selye H. The physiology and pathology of exposure to stress. Montreal: ACTA Inc.; 1950.

[44] McEwen BS. Central effects of stress hormones in health and disease: understanding the protective and damaging effects of stress and stress mediators. Eur J Pharmacol. 2008;583(2-3):174–85. 18282566 10.1016/j.ejphar.2007.11.071 PMC2474765

[45] Hayley S, Poulter M, Merali Z, Anisman H. The pathogenesis of clinical depression: stressor-and cytokine-induced alterations of neuroplasticity. Neuroscience. 2005;135(3):659–78. 16154288 10.1016/j.neuroscience.2005.03.051

[46] Pearlin LI. The life course and the stress process: some conceptual comparisons. J Gerontol - B Psychol Sci Soc Sci. 2010;65(2):207–15. 10.1093/geronb/gbp106 PMC2821941 20022925

[47] Sapolsky RM. Why zebras don’t get ulcers: The acclaimed guide to stress, stress-related diseases, and coping. Holt paperbacks; 2004.

[48] Turner RJ, Wheaton B, Lloyd DA. The epidemiology of social stress. Am Sociol Rev. 1995;60:104–25.

[49] Gold P, Chrousos G. Organization of the stress system and its dysregulation in melancholic and atypical depression: high vs low CRH/NE states. Mol Psychiatry. 2002;7(3):254–75. 11920153 10.1038/sj.mp.4001032

[50] Mello AF, Juruena MF, Pariante CM, Tyrka AR, Price LH, Carpenter LL, et al. Depression and stress: is there an endophenotype? Braz J Psychiatry. 2007;29:s13–8. 17546342 10.1590/s1516-44462007000500004 PMC4467732

[51] Heim C, Newport DJ, Mletzko T, Miller AH, Nemeroff CB. The link between childhood trauma and depression: insights from HPA axis studies in humans. Psychoneuroendocrinology. 2008;33(6):693–710. 18602762 10.1016/j.psyneuen.2008.03.008

[52] Harkness KL, Stewart JG, Wynne-Edwards KE. Cortisol reactivity to social stress in adolescents: role of depression severity and child maltreatment. Psychoneuroendocrinology. 2011;36(2):173–81. 20688438 10.1016/j.psyneuen.2010.07.006

[53] Van Veen T, Wardenaar K, Carlier I, Spinhoven P, Penninx B, Zitman F. Are childhood and adult life adversities differentially associated with specific symptom dimensions of depression and anxiety? Testing the tripartite model J Affect Disord. 2013;146(2):238–45. 23084183 10.1016/j.jad.2012.09.011

[54] Spinhoven P, Elzinga BM, Hovens JG, Roelofs K, Zitman FG, van Oppen P, et al. The specificity of childhood adversities and negative life events across the life span to anxiety and depressive disorders. J Affect Disord. 2010;126(1-2):103–12. 20304501 10.1016/j.jad.2010.02.132

[55] Gibb BE, Chelminski I, Zimmerman M. Childhood emotional, physical, and sexual abuse, and diagnoses of depressive and anxiety disorders in adult psychiatric outpatients. Depress Anxiety. 2007;24(4):256–63. 17041933 10.1002/da.20238

[56] Kendler KS, Myers J, Zisook S. Does bereavement-related major depression differ from major depression associated with other stressful life events? Am J Psychiatry. 2008;165(11):1449–55. 18708488 10.1176/appi.ajp.2008.07111757 PMC2743738

[57] Gold PW, Gabry KE, Yasuda MR, Chrousos GP. Divergent endocrine abnormalities in melancholic and atypical depression: clinical and pathophysiologic implications. Endocrinol Metab Clin. 2002;31(1):37–62. 10.1016/s0889-8529(01)00022-6 12055990

[58] Ouellet M-C, Savard J, Morin CM. Book review: insomnia following traumatic brain injury: A review. Neurorehabil Neural Repair. 2004;18(4):187–98. 15669131 10.1177/1545968304271405

[59] Metlaine A, Leger D, Choudat D. Socioeconomic impact of insomnia in working populations. Ind Health. 2005;43(1):11–9. 15732298 10.2486/indhealth.43.11

[60] Vitaro F, Brendgen M, Tremblay RE. Reactively and proactively aggressive children: antecedent and subsequent characteristics. J Child Psychol Psychiatry. 2002;43(4):495–505. 12030595 10.1111/1469-7610.00040

[61] Harkness KL, Monroe SM. Severe melancholic depression is more vulnerable than non-melancholic depression to minor precipitating life events. J Affect Disord. 2006;91(2-3):257–63. 16476487 10.1016/j.jad.2005.12.009

[62] Juruena MF, Bocharova M, Agustini B, Young AH. Atypical depression and non-atypical depression: is HPA axis function a biomarker? A systematic review. J Affect Disord. 2018;233:45–67. 29150144 10.1016/j.jad.2017.09.052

[63] Hill MN, Gorzalka BB. Is there a role for the endocannabinoid system in the etiology and treatment of melancholic depression?. Behav Pharmacol 2005;16(5-6):333–52. 16148438 10.1097/00008877-200509000-00006

[64] Gorman–Smith D, Tolan P. The role of exposure to community violence and developmental problems among inner-city youth. Dev Psychopathol. 1998;10(1):101–16. 9524810 10.1017/s0954579498001539

[65] Stephenson H, Pena-Shaff J, Quirk P. Predictors of college student suicidal ideation: gender differences. Coll Stud J. 2006;40(1):109.

[66] Depue RA, Monroe SM. Conceptualization and measurement of human disorder in life stress research: the problem of chronic disturbance. Psychol Bull. 1986;99(1):36. 3704034

[67] Trivedi MH, Rush A, Ibrahim H, Carmody T, Biggs M, Suppes T, et al. The inventory of depressive symptomatology, clinician rating (IDS-C) and self-report (IDS-SR), and the quick inventory of depressive symptomatology, clinician rating (QIDS-C) and self-report (QIDS-SR) in public sector patients with mood disorders: a psychometric evaluation. Psychol Med. 2004;34(1):73–82. 14971628 10.1017/s0033291703001107

