# Supplementary material for: Differential effects of lifetime stressors on major depressive disorder severity: a longitudinal community-based cohort study
Source: Eur Psychiatry. 2024 Oct 4;67(1):e66. doi: 10.1192/j.eurpsy.2024.1783 (PMC11536206; doi:10.1192/j.eurpsy.2024.1783)
Supplement: Su et al. supplementary material [file S0924933824017838sup001.docx]

| **Table S1.** Description of the study sample (N=1351). | |  |
| --- | --- | --- |
| Characteristics | N | Percentage |
| Sex |  |  |
| Male | 498 | 36.9% |
| Female | 853 | 63.1% |
| Age, n (%) |  |  |
| 18-29 years | 135 | 10.0% |
| 30-44 years | 364 | 26.9% |
| 45-59 years | 470 | 34.8% |
| 60 years and above | 382 | 28.3% |
| Marital status, n (%) |  |  |
| Single | 597 | 44.2% |
| Married/Common-law | 498 | 36.9% |
| Separated/Divorced/Widowed | 256 | 18.9% |
| Ethnicity, n (%) |  |  |
| White | 1179 | 87.3% |
| Non-white | 172 | 12.7% |
| Highest level of education, n (%) |  |  |
| Less than junior education | 92 | 6.8% |
| High school graduation | 375 | 27.8% |
| Post-high school | 884 | 65.4% |
| Immigration status |  |  |
| Yes | 250 | 19.1% |
| No | 1060 | 80.9% |
| Income |  |  |
| Less than $10,000 | 178 | 13.2% |
| $10,000 to $29,999 | 257 | 19.0% |
| $30,000 to $59,999 | 754 | 55.8% |
| $60,000 or more | 162 | 12.0% |
| Family history of mental disorders, n (%) |  |  |
| Yes | 562 | 41.6% |
| No | 789 | 58.4% |
| Comorbidities in mental illnesses |  |  |
| Yes | 846 | 62.6% |
| No | 505 | 37.4% |
